# Regulating the Hydrophilicity of Hyper-Cross-Linked Polymers via Thermal Oxidation for Atmospheric Water Harvesting

**DOI:** 10.1021/acsami.4c11013

**Published:** 2024-10-16

**Authors:** Paul Schweng, Lasse Präg, Robert T. Woodward

**Affiliations:** † Institute of Materials Chemistry and Research, Faculty of Chemistry, University of Vienna, Währinger Straße 42, 1090 Vienna, Austria; ‡ Vienna Doctoral School in Chemistry, University of Vienna, Währinger Straße 42, 1090 Vienna, Austria

**Keywords:** atmospheric water harvesting, hyper-cross-linked polymers, porous organic polymers, thermal oxidation, adsorption, direct air capture, hydrophilicity

## Abstract

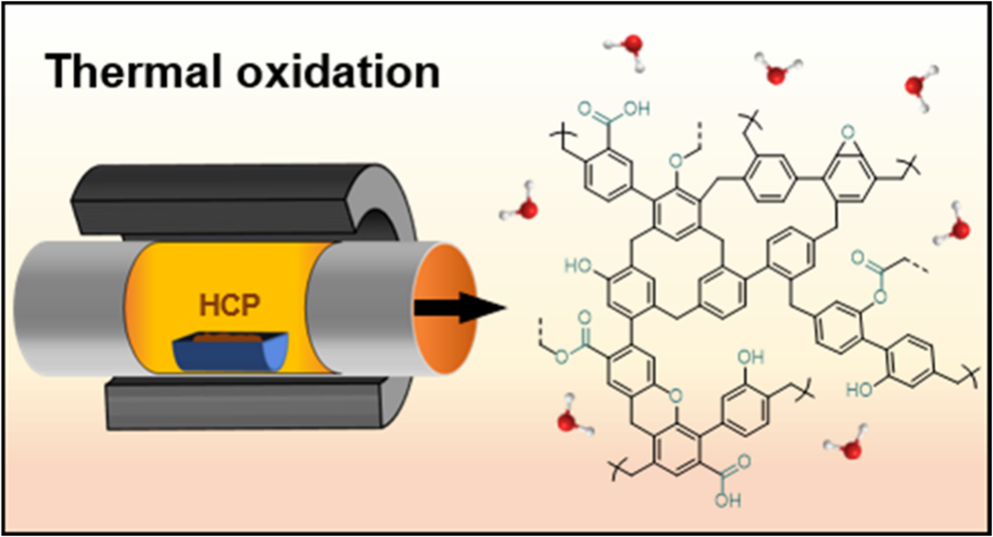

We explore the thermal oxidation of hyper-cross-linked polymers to enhance their hydrophilicity and efficacy in atmospheric water harvesting. Comprehensive chemical and physical characterizations are used to confirm the successful incorporation of polar oxygen moieties and the preservation of porosity upon thermal treatment. Newly introduced oxygen-based functional groups significantly improve water sorption properties, increasing total water uptake capacities by up to 400% and shifting water uptake onsets to significantly lower relative humidity. We also investigate the regeneration of oxidized hyper-cross-linked polymers after water sorption to probe their potential for multiple water harvesting cycles and reuse. Our findings outline a simple and cost-effective postsynthetic modification route for optimizing porous organic polymers for more sustainable and efficient atmospheric water harvesting.

## 1 Introduction

Access to clean water is a fundamental human right, underscored by the United Nations’ Sustainable Development Goal #6, which aims to ensure universal access to sanitation and clean water by 2030.^1^ In some regions, naturally occurring water sources such as rivers, lakes, and precipitation are insufficient to meet demand, leaving approximately one-third of the world’s population suffering from water scarcity, requiring supplementary water sources. Air contains an estimated 1.3 × 10^16^ L of water, vastly exceeding the volume of all of the world’s rivers combined.^2^ The extraction of water from air, known as atmospheric water harvesting (AWH), represents a promising geographically unrestricted solution for clean water production. Four primary methods are used for AWH: fog collection,^3^ refrigeration,^4^ membrane-separation,^5^ and adsorbent-based techniques. Adsorption-based systems show potential for water collection from low relative humidity (RH) environments by the passive capture and concentration of atmospheric water at their surfaces, from where it can be desorbed and harvested in a relatively energy-efficient process.^6^

Ideal adsorbents for AWH should possess high surface areas, hydrophilic sites, suitably low heats of adsorption, chemical and thermal stability, and fast adsorption–desorption kinetics for efficient water uptake and regeneration. Zeolites and hygroscopic metal salts are known for their remarkable total water uptake capacities, but the regeneration of the adsorbed water is typically energy-intensive. Metal–organic frameworks (MOFs) have emerged as promising candidates for AWH owing to their excellent uptake capacities and low heats of adsorption.^7^ However, many MOFs are water sensitive and degrade upon prolonged exposure to moisture, preventing long-term implementation and raising health concerns due to potential metal leaching.^8,9^

The search for AWH sorbents has recently focused on porous organic materials, which offer a broad array of synthetic routes, high surface areas, excellent stabilities, and a lack of requirement for metal constituents. Much research in this area is concentrated on covalent organic frameworks (COFs),^10^ reticular materials synthesized from various molecular organic building blocks bound together via reversible condensation reactions. The inclusion of hydrophilic moieties in ketoenamine-linked COFs, such as nitro, hydroxy, or sulfonate groups,^11,12^ led to significant water uptake at low RH compared to nonfunctionalized equivalents. Improvements in the hydrophilicity of azine-linked COFs were also achieved via the introduction of hydroxy groups, again shifting the water uptake onset to lower RH.^13^ Gruneberg et al. transformed imine-linkages to nitrone linkages in two COFs via oxidation, shifting the water uptake onset to lower RH, however, reducing the overall capacity.^14^ In another example, the oxidation of an azine-linked COF to hydrazides resulted in significantly improved water adsorption below 20% RH, without any reduction in total uptake capacity.^15^

Hyper-cross-linked polymers (HCPs) may represent a significant advancement in the field of AWH. These materials are densely cross-linked, micro/mesoporous networks produced via self-condensation reactions^16,17^ or by ’knitting’ together aromatic compounds using external cross-linkers.^18^ Typically, the synthesis of HCPs exploits Friedel–Crafts chemistry, allowing for the production of functional materials at a lower cost compared to many alternative porous organic polymers that rely on precious metal catalysts.^19^ We recently reported the first HCP for AWH, SHCP-10, a highly sulfonated network capable of adsorbing significant amounts of water at low relative humidity (0.22 g·g^–1^ at 30% RH) and displaying a total water uptake capacity of 0.81 g·g^–1^ at 25 °C.^20,21^ Another notable example of an amorphous porous organic polymer for AWH is an epoxy-functionalized network synthesized via a Diels–Alder reaction, which exhibited an overall capacity of 0.41 g·g^–1^ and excellent long-term stability.^22^ Due to the relative infancy of the field, amorphous micro/mesoporous polymers have yet to receive significant attention for AWH. We refer the reader elsewhere for an extensive list of amorphous micro/mesoporous polymers investigated for water uptake, although many were not explicitly reported for AWH.^23,24^

Oxidative degradation of polymers is a concern for their long-term application, with measures often implemented to mitigate this effect, such as the addition of antioxidants or avoiding exposure to UV light and/or heat. Thermogravimetric analysis (TGA) is employed to probe the thermal stability of porous polymers by heating the polymer under air flow and monitoring its weight over time. A slight weight increase attributed to oxidation is often observed in the temperature range 250–400 °C,^25^ prior to the onset of thermal decomposition.

In this work, HCPs are intentionally exposed to high temperatures in air with the aim of inducing thermal oxidation without significant degradation. This oxidation process introduces numerous oxygen-based polar moieties, which we anticipated would increase the hydrophilicity of the networks and improve their AWH performance. Our detailed examination of this process reveals a simple, inexpensive method to modify HCPs for improved water sorption. We provide an in-depth analysis of oxidized HCPs, focusing on their synthesis, characterization, and water adsorption properties. Comparison with nonoxidized equivalents highlights the advantages and potential applications of this approach.

## 2 Experimental Section

### 2.1 Materials

See the Supporting Information (SI).

### 2.2 Synthesis of Hyper-Cross-Linked Polymers

#### 2.2.1 Synthesis of Nonfunctionalized Biphenyl-Based Hyper-Cross-Linked Polymers (BP-HCPs)

Bis(chloromethyl)-1,1′-biphenyl (BCMBP, 7.5 mmol, 1.88 g) was dissolved in 1,2-dichloroethane (DCE, 10 mL) at room temperature over stirring. After dissolution, FeCl_3_ (11.25 mmol, 1.82 g) was added and a dark brown/black solid formed almost immediately. After 10 min at room temperature, a reflux condenser was attached to the flask and the mixture was heated to 80 °C for 22 h. After cooling, the formed solid was filtered and washed with methanol (100 mL) before being washed overnight via Soxhlet extraction, again with methanol. The resulting polymer was left in the fume cupboard for 3–4 h to remove excess methanol, before being lightly ground using a pestle and mortar and dried at 80 °C in a vacuum oven overnight. (Yield: 1.33 g, 99%).

#### 2.2.2 Preparation of Thermally Oxidized Hyper-Cross-Linked Polymers (OHCPs)

Around 0.5 g of BP-HCP was weighed into a ceramic boat and transferred into a tubular furnace (TG2 1200 °C Gradient equipped with a 3016 Controller, Carbolite Gero). The furnace temperature was ramped to 300 °C in air at a rate of 10 °C min^–1^ and then held at that temperature for either 5, 15, 30, or 60 min. It should be noted that the heating occurred in ambient air and no active air flow was used. The resulting networks were denoted as OHCP-X, where X denotes heating time at 300 °C.

### 2.3 Characterization

See the Supporting Information.

## 3 Results and Discussion

### 3.1 Characterization of Oxidized Hyper-Cross-Linked Polymers

Thermal oxidation is a postsynthetic modification and functionalization method used on a broad variety of materials, including MOFs,^26^ and porous organic polymers.^27^ Despite its simplicity and effectiveness, the optimization of annealing conditions, such as heat rates and isothermal heating times, is crucial to optimize the degree of oxidation without significant degradation of the network’s porous properties. To investigate this, we synthesized a nonfunctionalized HCP via the self-condensation of 4,4′-bis(chloromethyl)-1,1′-biphenyl for thermal oxidation. Owing to the biphenyl-based skeleton of the HCP, this nonfunctional network is named BP-HCP, herein. The detailed synthesis of BP-HCP is provided in the Experimental Section. The TGA of BP-HCP in air revealed a slight weight increase between 290 and 360 °C (Figure S1) and so this range was explored for the controlled thermal oxidation of the network.

We determined the optimal oxidation conditions for BP-HCP using TGA by heating to temperatures of either 280, 300, 320, or 340 °C for 60 min under air flow and monitoring any associated weight change (Table S1). Heat treatment at 280 °C led to a weight increase of 2.7 wt %, while treatment at 320 or 340 °C resulted in weight decreases due to thermal degradation. During heat treatment at 300 °C, we measured a weight increase of 7.3 wt % after 5 min, 8.7 wt % after 15 min, and reached a maximum of 9.0 wt % after 30 min. Heating for 60 min resulted in a slight weight reduction to a net weight gain of 8.1 wt %, likely due to some network degradation. As 300 °C treatment led to the most pronounced weight increase of BP-HCP, only this temperature was pursued further.

**Figure 1:**
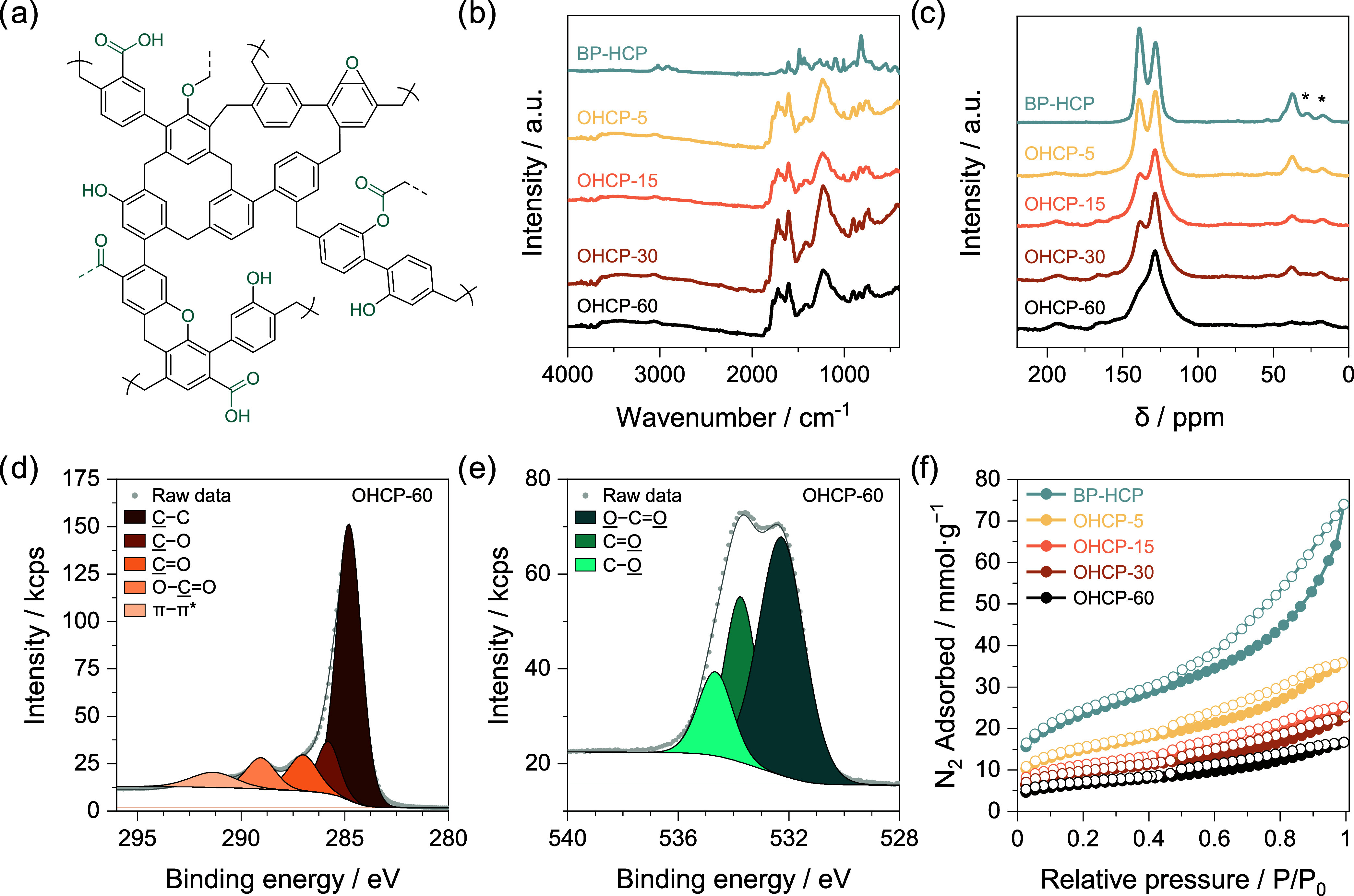
(a) Suggested representative structure for an OHCP. (b) FTIR spectra of BP-HCP and all OHCPs. (c) CP/MAS ssNMR spectra of BP-HCP and all OHCPs, * indicate spinning side bands. (d) X-ray photoelectron C 1s spectrum of OHCP-60. (e) X-ray photoelectron O 1s spectrum of OHCP-60. (f) N_2_ isotherms of BP-HCP and all OHCPs measured at −196 °C, filled and empty circles represent adsorption and desorption, respectively.

We used a tubular furnace to reproduce the results from our TGA experiments and explore the potential for scale-up. In brief, around 0.5 g of BP-HCP was ramped to 300 °C at a rate of 10 °C·min^–1^ and then held at that temperature for 5, 15, 30, or 60 min. The obtained polymers were named OHCP-X, where X denotes oxidation time at 300 °C in minutes. Photographs of BP-HCP and all OHCPs are shown in Figure S2. Based on the following chemical analyses, a suggested representative structure for an OHCP is shown in Figure 1a.

The successful oxidation of BP-HCP after heat treatment was confirmed using Fourier-transform infrared spectroscopy (FTIR, Figure 1b). All OHCP samples displayed a broad peak between 3600 and 3200 cm^–1^, regardless of oxidation time, corresponding to O–H stretching vibrations from alcohol groups. Characteristic signals for O–C=O stretching vibrations were observed at 1845, 1773, and 1717 cm^–1^, and are ascribed to the formation of anhydrides, phenyl esters and carboxylic acids, respectively. The presence of conjugated ketones was verified by the emergence of a peak at 1684 cm^–1^ corresponding to C=O and another at 1602 cm^–1^ assigned to C=C stretching vibrations. Characteristic signals for aryl ethers were observed at 1231 cm^–1^.

To verify the presence of oxygen functionalities in OHCPs, we employed ^13^C cross-polarization/magic angle spinning solid-state NMR (CP/MAS ssNMR) (Figure 1c). The spectra revealed weak signals at ∼219, ∼193, ∼180 and ∼166 ppm, ascribed to newly formed ketone, aldehyde, carboxylic acid and ester groups, respectively. Peaks at ∼156 ppm and ∼78 ppm indicated the presence of phenolic and aliphatic C–O bonds, while a weak signal at ∼42 ppm was assigned to C–Cl. As oxidation time increased, we observed a notable rise in the intensity of peaks associated with oxygen-bound carbons, confirming greater oxygen incorporation. Conversely, the intensity of signals at ∼128, ∼138 ppm, and ∼37 ppm, corresponding to aromatic carbon (C_Ar_–H), substituted aromatic carbon (C_Ar_–R), and methylene bridges, respectively, decreased with heating time. This decrease may be due to the downfield shift of carbon signals upon oxidation and to some framework degradation under prolonged heat exposure, in good agreement with initial TGA experiments.

We performed X-ray photoelectron spectroscopy (XPS) to gain an in-depth understanding of the chemical composition of OHCPs. The high-resolution C 1s spectrum of OHCP-60 (Figure 1d) displayed a predominant feature at a binding energy of 284.8 eV, attributed to C–C bonding, encompassing both sp2 aromatic carbon and sp3 carbon present in methylene bridges. A peak at 286.0 eV in the shoulder of the main component is assigned to C–O, arising from the incorporation of alcohols, ethers, peroxides, and epoxides. The presence of ketone and aldehyde functionalities is confirmed by the emergence of a peak at 287.1 eV, corresponding to C=O bonding. The peak at a binding energy of 288.6 eV is attributed to O–C=O moieties originating from carboxylic acids, esters, and anhydrides. A broad π–π* shakeup feature of low intensity is also observed at 290.7 eV. The high-resolution O 1s spectrum of OHCP-60 (Figure 1e), confirmed the presence of O–C=O with a peak at a binding energy of 532.4 eV, as well as of C=O and C–O with peaks at 533.8 and 534.6 eV, respectively. With longer heating times, the oxygen content increased from 16.34 ± 0.01 wt % after 5 min to 27.24 ± 0.30 wt % after 60 min at 300 °C. High-resolution C 1s and O 1s spectra are provided for all remaining OHCPs in the SI (Figure S3) and the surface chemical composition of all HCPs is provided in Table S2. Using the carbon mass of all polymers determined by elemental analysis (Table S3), the oxidation efficiency η was calculated to be 6%, 11%, 12%, and 17% for OHCP-5, OHCP-15, OHCP-30, and OHCP-60, respectively. The calculation of η is described in the SI.

We performed N_2_ sorption analysis on all HCPs at −196 °C to monitor the change in textural properties upon oxidation (Figure 1f, Table 1). All networks, including pristine BP-HCP, displayed features of both type I and type IVa isotherms, indicative of micropores and meso-/macropores, respectively.^28^ All HCPs exhibited H3 hysteresis curves, signifying disordered pore structures and wide pore size distribution. Hysteresis became less pronounced with increasing oxidation time, suggesting a reduction in the mesopore content of the networks. This reduction is also reflected in a gradual decrease in BET specific surface area, SSA_BET_, and total pore volume, *V*_TOT_, with oxidation time from 1770 ± 47 m^2^·g^–1^ and 2.12 ± 0.14 cm^3^·g^–1^ for BP-HCP to 462 ± 68 m^2^·g^–1^ and 0.52 ± 0.07 cm^3^·g^–1^ in OHCP-60.

**Table 1:** Summary of the Porous Properties and Water Sorption of BP-HCPs and All OHCPs, Including BET Specific Surface Area, SSA_BET_, Micropore Volume,*V*_MICRO_, Total Pore Volume,*V*_TOT_, and Water Uptake at 10, 30, and 90% RH at 25 °C^a^

				water uptake (g·g^–1^)		
sample	SSA_BET_ (m^2^·g^–1^)	*V*_MICRO_ (cm^3^·g^–1^)	*V*_TOT_ (cm^3^·g^–1^)	10% RH	30% RH	90% RH
BP-HCP	1770 ± 47	0.17 ± 0.04	2.12 ± 0.14	0.00	0.00	0.08 ± 0.01
OHCP-5	1029 ± 127	0.15 ± 0.01	1.05 ± 0.15	0.01 ± 0.00	0.03 ± 0.01	0.38 ± 0.02
OHCP-15	860 ± 152	0.14 ± 0.03	0.89 ± 0.14	0.02 ± 0.00	0.05 ± 0.01	0.37 ± 0.01
OHCP-30	681 ± 109	0.10 ± 0.01	0.73 ± 0.12	0.02 ± 0.00	0.04 ± 0.01	0.35 ± 0.02
OHCP-60	462 ± 68	0.06 ± 0.02	0.52 ± 0.07	0.03 ± 0.01	0.06 ± 0.01	0.35 ± 0.01

a Data are averaged from a sample number, *n*, of 3 (*n* = 3).

We analyzed the thermal stability of all OHCPs after heat treatment using TGA in air (Figure S4). Negligible weight loss was observed until >350 °C, confirming high thermal stability of the networks after oxidation. Weight loss prior to 110 °C increased with extended oxidation time, reaching a maximum of 5 wt % for OHCP-60, indicative of elevated water adsorption at higher oxidation densities. Unlike the pristine sample, no weight increase was observed in the OHCPs at around 300 °C, indicative of no significant further oxidation.

### 3.2 Water Sorption Experiments

**Figure 2:**
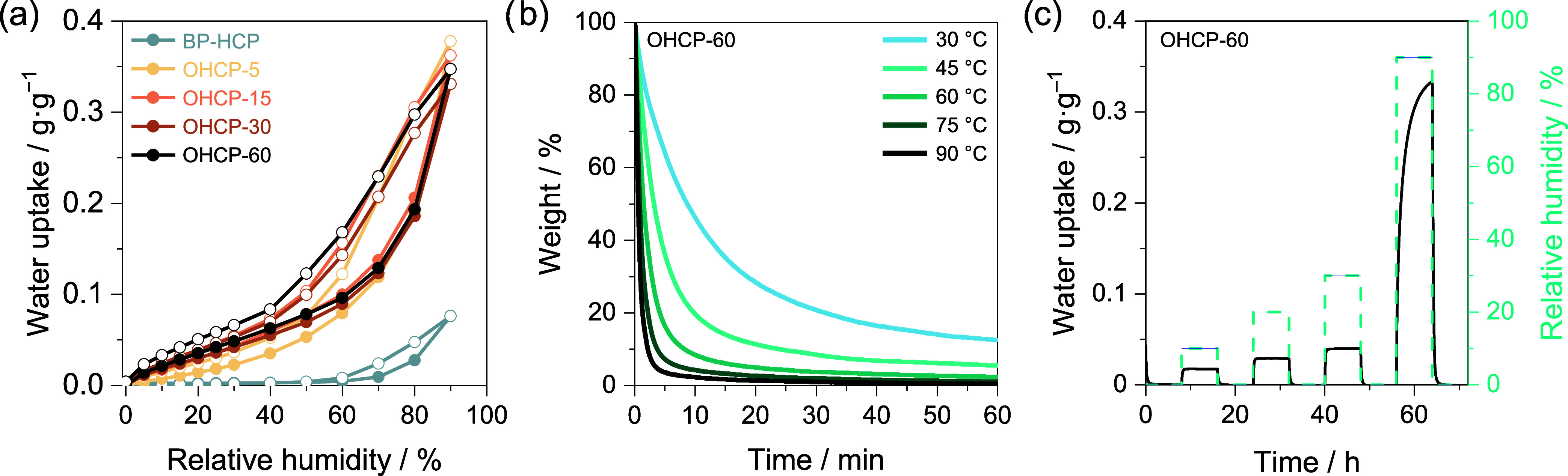
Water sorption–desorption properties of BP-HCP and OHCPs. (a) Water isotherms at 25 °C. Closed spheres represent uptake and open spheres represent desorption. (b) Desorption of water from OHCP-60 over time at various temperatures, measured using TGA. (c) Water sorption–desorption of OHCP-60 at 10%, 20%, 30%, and 90% RH.

The capability of OHCPs to adsorb water from air was assessed by measuring water sorption isotherms using dynamic vapor sorption (DVS, Figure 2a, raw data provided in Figure S5). Pristine BP-HCP reached a total water sorption capacity of just 0.07 g·g^–1^ at 90% RH and 25 °C. In comparison, the water uptake onset of all OHCPs was shifted to lower RH and the total water capacity was increased. Network OHCP-5 displayed the highest total water uptake (0.38 g·g^–1^ at 90% RH and 25 °C) due to the coupling of increased hydrophilicity upon oxidation and retention of high surface area and total pore volume. The water sorption capacity within the critical range of <30% RH improved across all samples upon oxidation, with the highest enhancement observed in OHCP-60, achieving a water adsorption capacity of 0.05 g·g^–1^ at 30% RH and 0.03 g·g^–1^ at 10% RH, confirming the role of newly introduced hydrophilic oxygen moieties. The superior water uptake of OHCP-60 at low RH, in contrast to its inferior porous properties, is attributed to the high concentration of hydrophilic oxygen moieties introduced during oxidation, consistent with our previous findings.^23^ Careful control of the chemical and textural properties of OHCPs provides valuable insight into the characteristics required for AWH. Hydrophilic moieties are critical for uptake at low RH, but surface area plays a more important role in the overall uptake capacity once sufficient hydrophilicity is achieved. Here, OHCP-60 was selected as a representative material due to its performance at low RH, despite possessing a lower total water adsorption capacity than the other OHCPs. Nevertheless, the water sorption and porous properties of all networks are summarized in Table 1 and compared with examples from the literature in Table S4.

We exposed OHCPs to ∼45% RH and 20 °C for 24 h to achieve water loading. Water-loaded specimens were then subjected to temperatures of 30, 45, 60, 75, and 90 °C in a TGA under dry air flow and water desorption was measured gravimetrically (OHCP-60 shown in Figure 2b). Water release was accelerated with increasing temperature, leading to increased removal of the adsorbed water. The time required to reach desorption equilibrium increased gradually with oxidation time (Figure S6), possibly due to stronger water-network interactions with increasing oxygen content. Water removal of >90% was achieved for all samples within 10 min at 60 °C, and >98% over the same time at 90 °C.

We subjected OHCPs to 10, 20, 30, and 90% RH at 25 °C, and monitored water uptake gravimetrically to gain a deeper understanding of the rate of adsorption/desorption. Following 8 h exposure to 30% RH, OHCP-60 exhibited a water sorption capacity of 0.05 g·g^–1^ (Figure 2c). Remarkably, at ≤30% RH complete water uptake occurred within 30 min, representing an improvement over SHCP-10,^20^ which reached its total capacity in ∼1 h under the same conditions. It should be noted, however, that the uptake capacity of SHCP-10 in this range remains much higher (0.22 g·g^–1^). Uptake and desorption experiments for all OHCPs are shown in Figure S7. The water diffusivity of OHCP-60 was estimated to be 1.53 × 10^–14^ m^2^·s^–1^ by fitting the uptake measurements at 30% RH and 25 °C to Fick’s laws of diffusion.^29^

**Figure 3:**
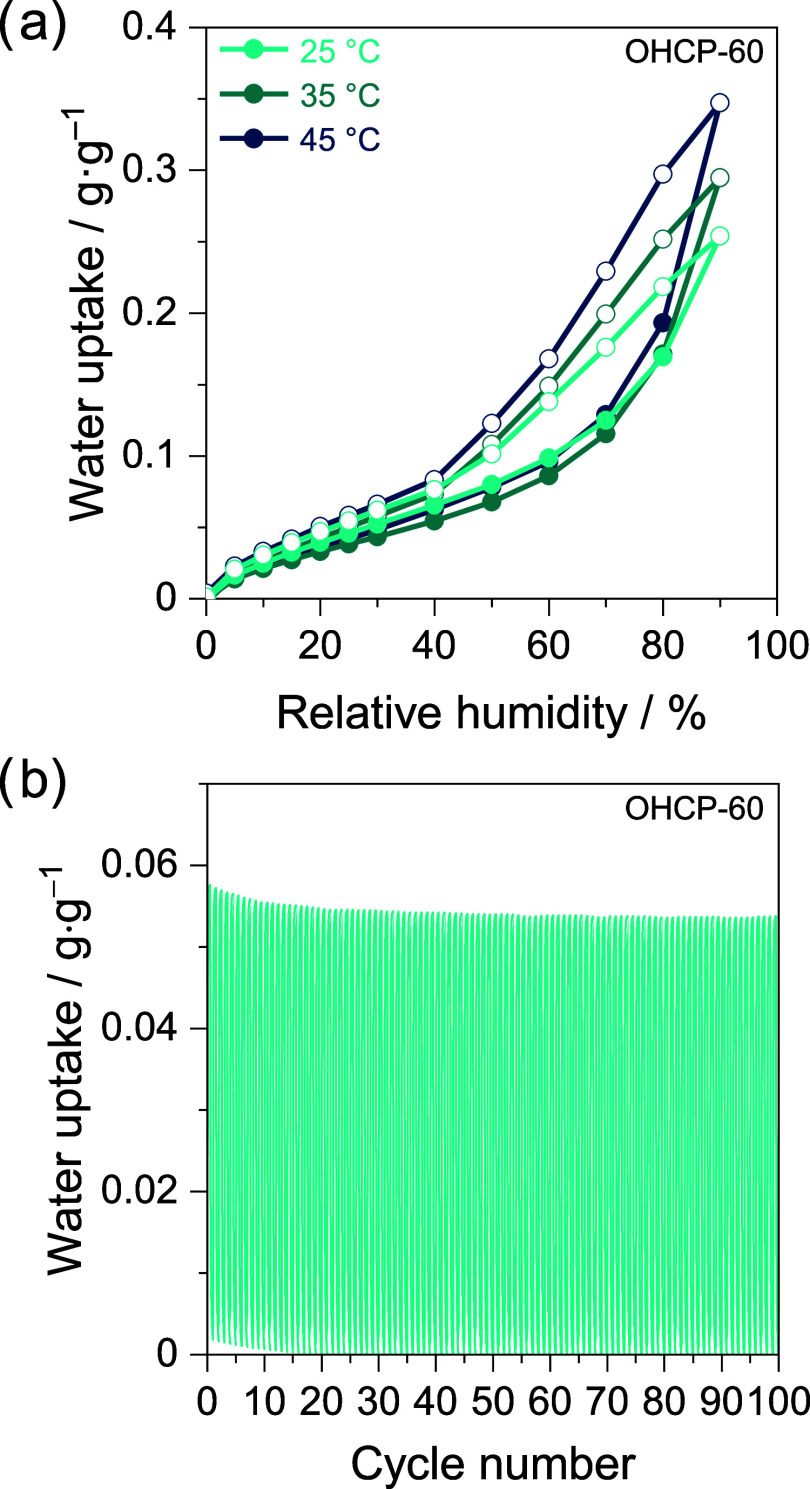
(a) Water sorption–desorption isotherms of OHCP-60 recorded at 25, 35, and 45 °C. (b) Long-term stability of OHCP-60 over 100 adsorption–desorption cycles consisting of a humidity-swing process between 0% and 40% RH.

Additional water sorption isotherms for sample OHCP-60 at 35 and 45 °C (Figure 3a) revealed that water uptake was retained at elevated temperatures, highlighting robust water sorption. Using the Clausius–Clapeyron equation on the 25 and 35 °C water sorption isotherms, we determined the isosteric heat of adsorption (*Q*_st_) to be 43 kJ·mol^–1^, a similar value to that of bulk water (*Q*_st_ = 44 kJ·mol^–1^). The *Q*_st_, being similar to the condensation enthalpy of water, suggests that water–water interactions are predominant during pore-filling, rather than water-network interactions.

**Figure 4:**
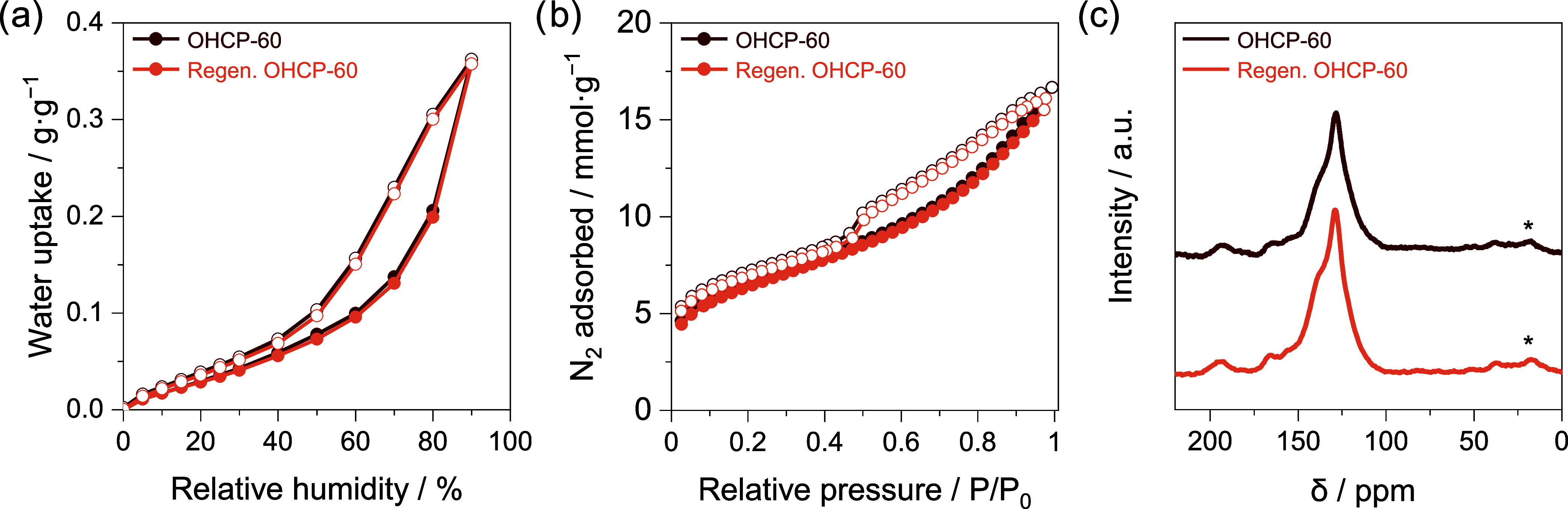
Comparison of OHCP-60 (brown) and OHCP-60 after 10 sorption–desorption cycles (orange), in which desorption was driven by heating at 90 °C for 1 h per cycle. (a) Water isotherms measured at 25 °C, (b) N_2_ isotherms measured at −196 °C. Filled and empty circles represent adsorption and desorption, respectively, and (c) CP/MAS ssNMR spectra, where * indicate spinning side bands.

Lastly, we probed the long-term stability of OHCPs over numerous water adsorption–desorption cycles using either heat or a reduced RH as desorption trigger. OHCP-60 exhibited a negligible reduction in water uptake over 100 humidity swings between 0 and 40% RH (Figure 3b), highlighting the potential of OHCPs for long-term AWH. We further explored the stability of OHCPs by reanalysis after a total of 10 adsorption–desorption cycles using heat as a trigger for water removal (90 °C for 1 h in each cycle), as we deemed this a harsher desorption method. After the heat swings, the water sorption isotherm of OHCP-60 at 25 °C (Figure 4a) exhibited negligible decrease in water uptake capacity. Postcycling, N_2_ sorption isotherms of OHCPs revealed no measurable change in textural properties (OHCP-60 shown in Figure 4b, data for all remaining OHCPs is provided in Figure S8 and Table S5). Furthermore, no chemical change was observed in regenerated OHCP-60 when compared to the pristine network (Figure 4c). The integrity of all OHCPs was confirmed by further chemical and physical characterization including FTIR, TGA, and ssNMR (Figures S9–S11), revealing no measurable difference. The retention of the chemical and physical properties of OHCPs upon heat cycling underpins the excellent stability of OHCPs for AWH.

## 4 Conclusions

In this study, we have demonstrated that thermal oxidation of a hyper-cross-linked polymer, BP-HCP, at 300 °C significantly enhances its water sorption capability, rendering them effective materials for atmospheric water harvesting (AWH). Compared to nonfunctional BP-HCP, oxidized HCPs (OHCPs) exhibited both superior total water uptake capacities and adsorption at low relative humidity. Network OHCP-5 showed a total water capacity of 0.38 g·g^–1^ at 90% RH and OHCP-60 demonstrated good performance in AWH-relevant conditions, with 0.05 g·g^–1^ at 30% RH and 25 °C. The water uptake performance of OHCP-60 at low RH, despite having the lowest pore volume and surface area, was attributed to the introduction of high levels of hydrophilic oxygen-based moieties. Longer thermal oxidation times led to improved water uptake in low RH regimes but reduced total uptake capacities due to inferior porous properties. Postsorption analyses indicated no significant chemical or physical changes in OHCPs after multiple water sorption–desorption cycles, demonstrating their recyclability and potential for continued reuse. These findings suggest that simple and cost-effective thermal oxidation can be utilized to optimize HCPs, or porous organic polymers more broadly, for sustainable AWH applications, offering a promising solution for addressing global water scarcity challenges.

## Supporting Information Available

Materials and characterization details. TGA for thermal oxidation, thermal stability of OHCPs, and water desorption experiments, HCP photographs, XPS, elemental analysis, comparison of OHCPs with leading porous organic polymers for AWH, and DVS for water sorption upon changes in RH. Analysis of regenerated OHCPs is also provided, including N_2_ sorption–desorption isotherms, porous properties, FTIR spectra, ssNMR spectra, and TGA (PDF)
