# Supplementary material for: Regulating the Hydrophilicity of Hyper-Cross-Linked Polymers via Thermal Oxidation for Atmospheric Water Harvesting
Source: ACS Appl Mater Interfaces. 2024 Oct 16;16(43):58566–72. doi: 10.1021/acsami.4c11013 (PMC11533164; doi:10.1021/acsami.4c11013)
Supplement: Supplementary file 1 — am4c11013_si_001.pdf [file am4c11013_si_001.pdf]

Supporting Information

Regulating hydrophilicity of hypercrosslinked polymers via thermal oxidation for atmospheric water harvesting

Paul Schweng,[a,b] Lasse Präg,[a] Robert T. Woodward[a]*

[a] Institute of Materials Chemistry and Research, Faculty of Chemistry, University of Vienna, Währinger Straße 42, 1090, Vienna, Austria

[b] Vienna Doctoral School in Chemistry, University of Vienna, Währinger Straße 42, 1090, Vienna, Austria

*Corresponding author:

E-mail: robert.woodward@univie.ac.at

1. Experimental

1.1. Materials

All chemicals were purchased from commercial suppliers and used as received. 4,4´-Bis(chloromethyl)-1,1´-biphenyl (BCMBP, 95 %), iron(III)chloride ($FeCl_3$, 97 %) and 1,2-dichloroethane (DCE, ≥ 99 %) were purchased from Sigma-Aldrich. Methanol (≥99.8%) was purchased from Fisher Scientific.

1.2. Characterisation of hypercrosslinked polymers

Fourier-transform infrared spectroscopy (FTIR) was performed using a Tensor II FTIR spectrometer (Bruker) equipped with a Bruker Optics Platinum ATR module. Measurements were performed on finely ground samples in ambient conditions. The IR spectra were obtained in a double-sided forward–backward acquisition mode in the range of 400–4000 cm^{-1} with a spectral resolution of 4 cm^{-1}. Spectra were obtained by averaging a total of 32 scans and were calculated using a Blackman–Harris 3-term apodization function and a zero-filling factor of 4. The instrument was flushed with dry air during the measurement, and the spectra were recorded and analysed using the software OPUS 7.5.

X-ray photoelectron spectroscopy (XPS) was performed on a Nexsa Photoelectron Spectrometer (Thermo Scientific). High-resolution spectra of carbon (C 1s 279–298 eV) and oxygen (O 1s 525–545 eV) were recorded with a resolution of 0.1 eV and a pass energy of 50 eV. All measurements were performed using Al-Kα X-rays with a spot size of 400 µm. Evaluation of the spectra was performed using Avantage software (v5.9931, Thermo Fisher Scientific) and the atomic composition of the sample was determined from the peak area using the integrated scaling factor database ALTHERMO1. Data was averaged over a total of three measurements (n = 3).

Elemental analysis was performed using a Eurovector EA 3000 CHNS-O Elemental Analyser. Up to 3.0 mg of sample was weighed via micro balance (Sartorius, ME 5 OCE) into tin vials (4×6 mm) and measured at least in triplicate. The operating temperatures for the combustion and reduction were 1000 °C (1480 °C for O analysis) and 750 °C, respectively, with helium (99.999+) used as carrier gas. Oxygen efficiency η was calculated using the following equation:

$$\eta = \frac{C_{in} - C_{fin}}{C_{in}} \cdot 100\%$$

where C_{in} is the initial carbon mass in g and C_{fin} the mass of carbon in g after thermal oxidation.

Thermogravimetric analysis was performed using a Discovery TGA from TA instruments (New Castle, DE, United States). For thermal stability experiments, approximately 10 mg of sample was heated under dry air flow (100 $mL \cdot min^{-1}$) at a rate of 10 °C min^{-1} from room temperature to 700 °C. For thermal oxidation experiments, approximately 10 mg of sample was heated under dry air flow (100 $mL \cdot min^{-1}$) at a rate of 10 °C min^{-1} from room temperature to 280, 300, 320, or 340 °C and held at the target temperature for 60 min before cooling.

Nitrogen adsorption-desorption isotherms were recorded at –196 °C (77 K) using a TriStar II (Micromeritics Instrument Corporation). Samples were degassed for at least 4 h at 120 °C under N_2 flow using a FlowPrep 060 (Micromeritics Instrument Corporation). Sample surface areas were calculated using the Brunauer-Emmett-Teller (BET) method on the adsorption branch between 0.05 - 0.2 P/P_0. The total pore volume was calculated from the volume of N_2 adsorbed at P/P_0 = 0.97 and the micropore volume was determined using the t-plot method between 0.15 – 0.4 P/P_0. Data was averaged over a total of three measurements (n = 3).

1.3. Water sorption experiments using thermally oxidised HCPs

Dynamic vapour sorption (DVS) experiments were performed using a DVS-Resolution (Surface Measurement Systems). Approximately 10 mg of sample was weighed into a quartz crystal pan. Deionised water was used to generate the desired RH. Measurements began at 0% RH to

removal residual water before being increased to the desired RH using deionised water. Experiments were carried out at 25 °C, unless stated otherwise. Isotherms were recorded up to 90% RH, using a step increment of 5% RH. Each step was equilibrated for 5 h, prior to measurement. To assess the water diffusivity, the water uptake data of OHCP-60 at 30% RH and 25 °C was fitted to Fick's law of diffusion using the integrated software provided by Surface Measurement Systems.

Water desorption experiments were performed on a Discovery TGA (TA instruments). Samples were conditioned at ~45% RH and 20 °C for 24 h to achieve water loading. Subsequently, around 20 mg of conditioned sample was transferred to the TGA and held at a constant temperature of either 30, 45, 60, 75, and 90 °C for 1 h under dry air flow (25 $mL \cdot min^{-1}$) and monitored gravimetrically.

2. Supporting figures and tables

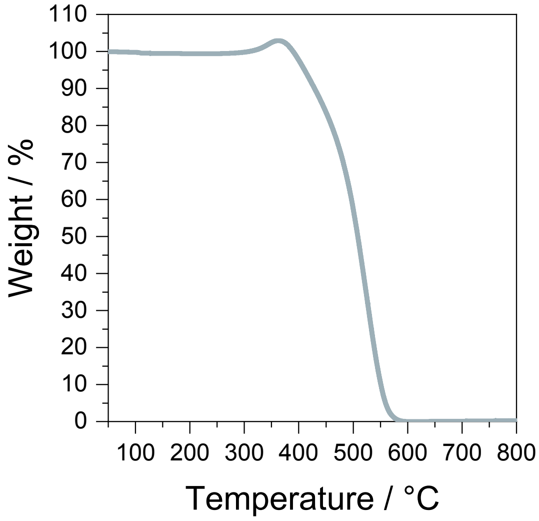

Figure S1. TGA of BP-HCP heated under airflow (100 mL·min^{-1}) to 800 °C at a rate of 10 °C/min.

Table S1. Weight change of BP-HCP upon thermal oxidation determined using TGA for a variety of temperatures and hold times.

Hold time at 300 °C (min)	Weight change (wt.%)			
	280 °C	300 °C	320 °C	340 °C
5	-0.5	7.3	1.7	2.8
15	0.4	8.7	2.3	1.1
30	1.6	9.0	1.9	-2.0
60	2.7	8.1	-0.5	-8.0

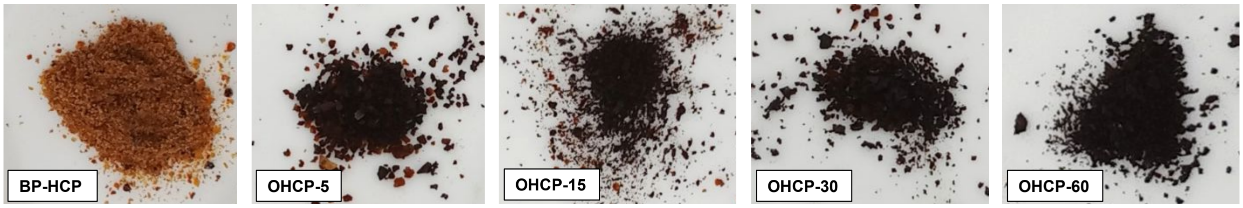

Figure S2. Photographs of BP-HCP and all OHCPs.

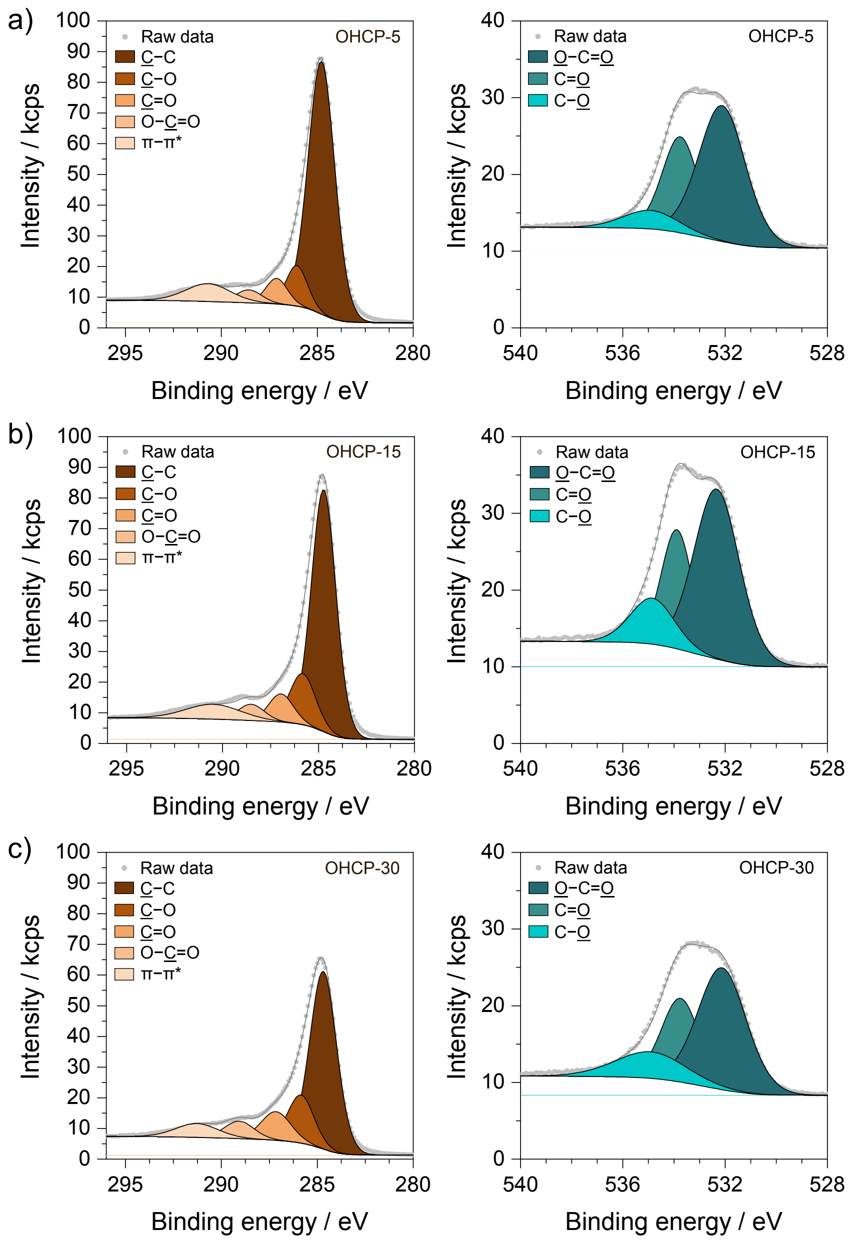

Figure S3. X-ray photoelectron C 1s and O 1s spectra of a) OHCP-5, b) OHCP-15, and c) OHCP-30.

Table S2. Elemental composition of BP-HCP and all OHCPs determined by XPS (n=3).

	C (wt.%)	N (wt.%)	O (wt.%)	Cl (wt.%)	S (wt.%)	O (mmol·g^{-1})
BP-HCP	92.57 ± 0.52	0.14 ± 0.11	2.57 ± 0.28	4.31 ± 0.14	0.14 ± 0.11	1.61 ± 0.18
OHCP-5	79.98 ± 0.35	0.11 ± 0.01	16.34 ± 0.01	3.55 ± 0.35	0.03 ± 0.02	10.21 ± 0.01
OHCP-15	77.37 ± 0.33	0.10 ± 0.03	20.38 ± 0.40	2.11 ± 0.01	0.06 ± 0.05	12.74 ± 0.25
OHCP-30	74.66 ± 0.97	0.07 ± 0.01	22.48 ± 0.34	1.84 ± 0.29	0.05 ± 0.01	14.05 ± 0.21
OHCP-60	71.72 ± 0.10	0.10 ± 0.01	27.24 ± 0.30	0.88 ± 0.25	0.07 ± 0.01	17.02 ± 0.19

Table S3. Elemental composition of BP-HCP and all OHCPs determined by elemental analysis (n=3).

	C (wt.%)	H (wt.%)	N (wt.%)	S (wt.%)	O (wt.%)	O (mmol·g^{-1})
BP-HCP	85.70 ± 0.21	4.96 ± 0.03	< 0.05	< 0.02	2.84 ± 0.33	1.78 ± 0.21
OHCP-5	79.72 ± 1.07	4.08 ± 0.07	< 0.05	< 0.02	13.24 ± 0.72	8.28 ± 0.45
OHCP-15	75.14 ± 1.15	3.45 ± 0.09	< 0.05	< 0.02	20.81 ± 0.99	13.01 ± 0.62
OHCP-30	73.89 ± 0.54	3.51 ± 0.16	< 0.05	< 0.02	22.35 ± 1.38	13.97 ± 0.86
OHCP-60	71.27 ± 0.69	3.07 ± 0.10	< 0.05	< 0.02	25.76 ± 0.96	16.10 ± 0.60

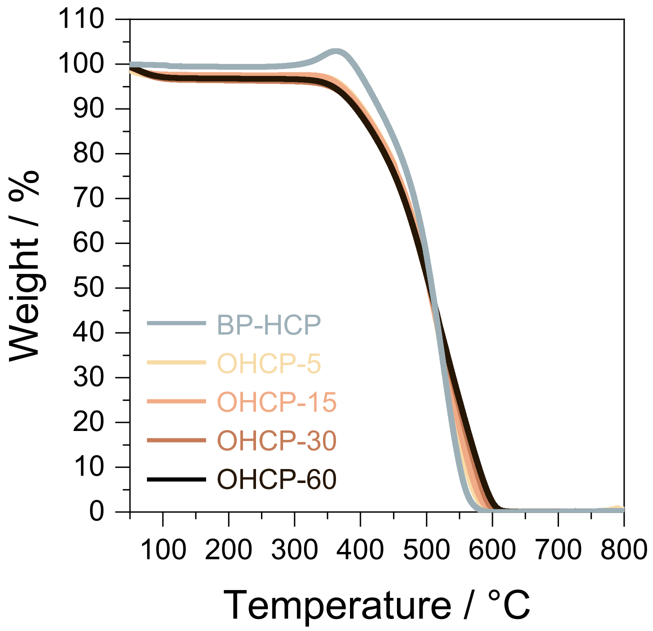

Figure S4. TGA of BP-HCP and OHCPs heated under airflow (100 mL·min^{-1}) to 800 °C at a rate of 10 °C/min.

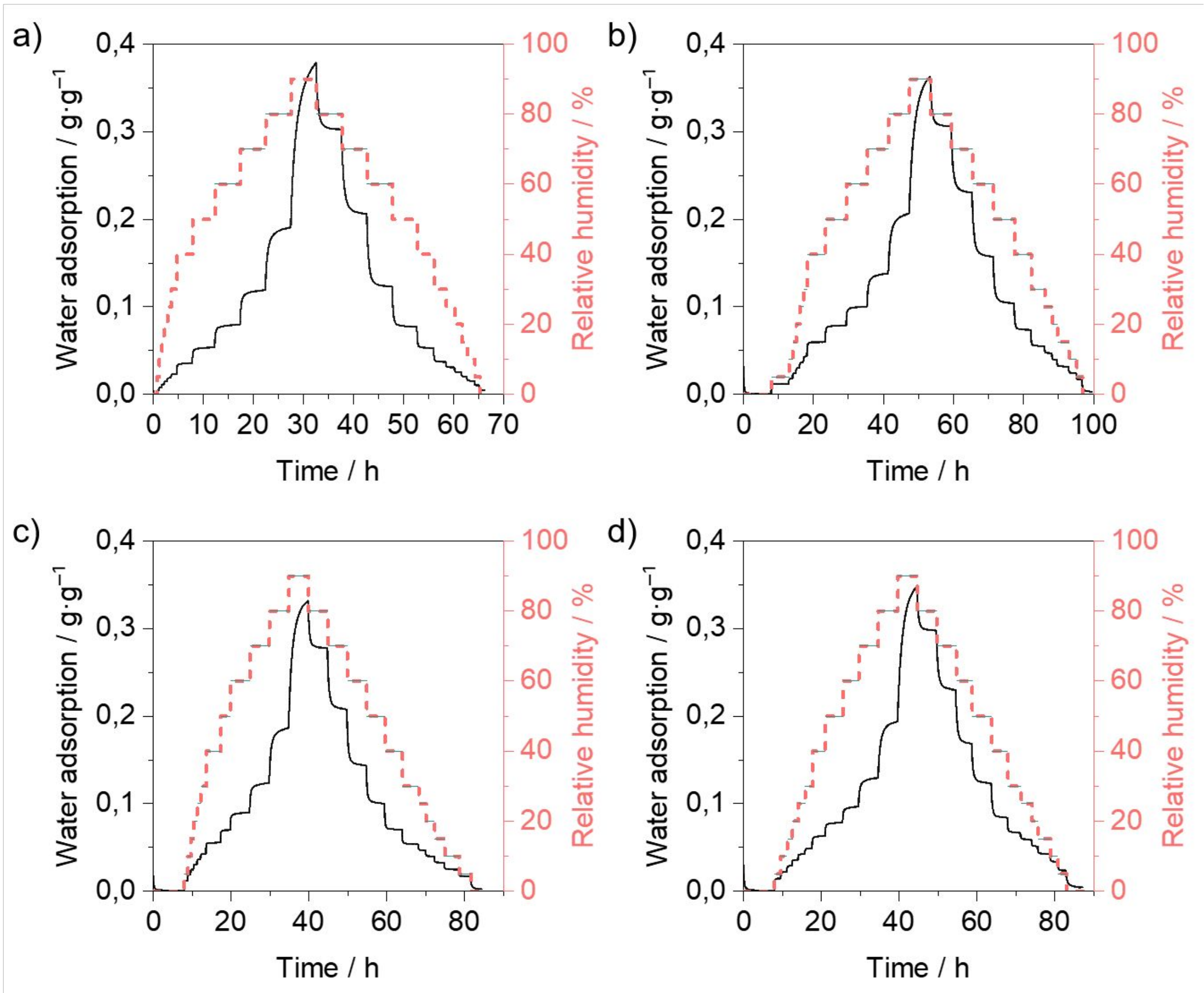

Figure S5. Water sorption over time with increasing and subsequent decreasing RH. Water sorption isotherms were produced using these data sets by recording weight gain/loss after equilibration at a given RH. a) OHCP-5, b) OHCP-15, c) OHCP-30, and d) OHCP-60

Table S4. Summary of network chemistry, BET surface area, water vapour uptake at 0.3 P/P_0 (30% RH), and total uptake capacity of various porous organic sorbents for water sorption.

Material	Network chemistry	BET surface area [m^2/g]	H_2O uptake at 0.3 P/P_0 [g/g]	Total uptake capacity [g/g]	Uptake T [°C]	Ref.
**Covalent Organic Frameworks**						
TpBD	Ketoenamine	341	0.02	0.15	25	1
TpBD-Me_2	Ketoenamine / $-CH_3$	-	0.02	0.14	25	1
TpBD-$(OMe)_2$	Ketoenamine / $-OCH_3$	365	0.02	0.16	25	1
TpBD-$(NO_2)_2$	Ketoenamine / $-NO_2$	90	0.02	0.09	25	1
COF-SO_3H	Ketoenamine / $-SO_3H$	280	0.19	0.31	25	2
SHTA-Pa	Ketoenamine / $(Enamine)_2$	1848	0.01	0.45	25	3
DHTA-Pa	$(Ketoenamine)_2$ / Enamine	2099	0.48	0.65	25	3
THTA-Pa	Ketoenamine	772	0.21	0.33	25	3
COF-ok	Ketoenamine / Pyrene	1194	0.20	0.64	25	4
Py-MPA	Imine / Pyrene	604	0.01	0.09	25	5
Py-PDCA	Imine / Pyrene / Pyridine	643	0.02	0.18	25	5
Py-HMPA	Imine / Pyrene / –OH	705	0.07	0.23	25	5
COF-432	Imine	895	0.01	0.30	25	6
3D-CageCOF-1	Imine / Phenyl ether	1040	0.16	0.30	25	7
PI-3-COF	Imine / Triazine	1340	0.03	0.46	25	8
NO-PI-3-COF	Nitrone / Triazine	664	0.15	0.24	25	8
AB-COF	Hydrazine	1209	0.33	0.44	25	9
COF-480-hydrazide	Hydrazide	989	0.32	0.44	25	9
AB-COF	Azine	1125	0.30	0.41	25	10
ATFG-COF	Keto-Enol tautomer	520	0.14	0.25	25	10
g-DZPH-COF	Vinylene / Pyridazine	960	0.39	1.00	25	11
g-DZTA-COF	Vinylene / Pyridazine / Triazine	802	0.07	0.99	25	11
**Covalent Triazine Frameworks**						
bpim-CTF-400	Bis(pyridyl) imidazolium	786	0.22	0.38	25	12
bpim-CTF-500	Bis(pyridyl) imidazolium	1556	0.16	0.58	25	12
FJU-CTF-FIZ500	Benzimidazole	2042	0.21	0.82	23	13
FJU-CTF-TIZ500	Benzimidazole	1732	0.21	0.60	23	13
**Amorphous micro-/mesoporous POPs**						
SHCP-10	$-SO_3H$	697	0.22	0.81	25	14
2D ep-POP	Oxirane (epoxide)	852	0.10	0.41	25	15
3D ep-POP	Oxirane (epoxide)	779	0.18	0.41	25	15
OHCP-5	Thermal oxidation	1029	0.03	0.38	25	This work
OHCP-15	Thermal oxidation	860	0.05	0.37	25	This work
OHCP-30	Thermal oxidation	681	0.04	0.35	25	This work
OHCP-60	Thermal oxidation	462	0.06	0.35	25	This work

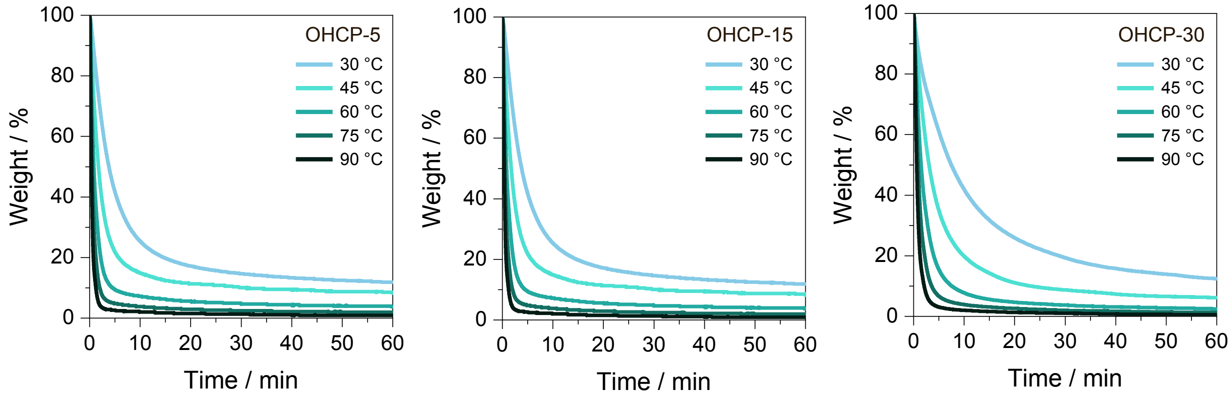

Figure S6. Water desorption from OHCP-5, OHCP-15, and OHCP-30, measured using TGA. Desorption was performed at a variety of temperatures after networks were conditioned at ~45% RH and 20 °C, overnight.

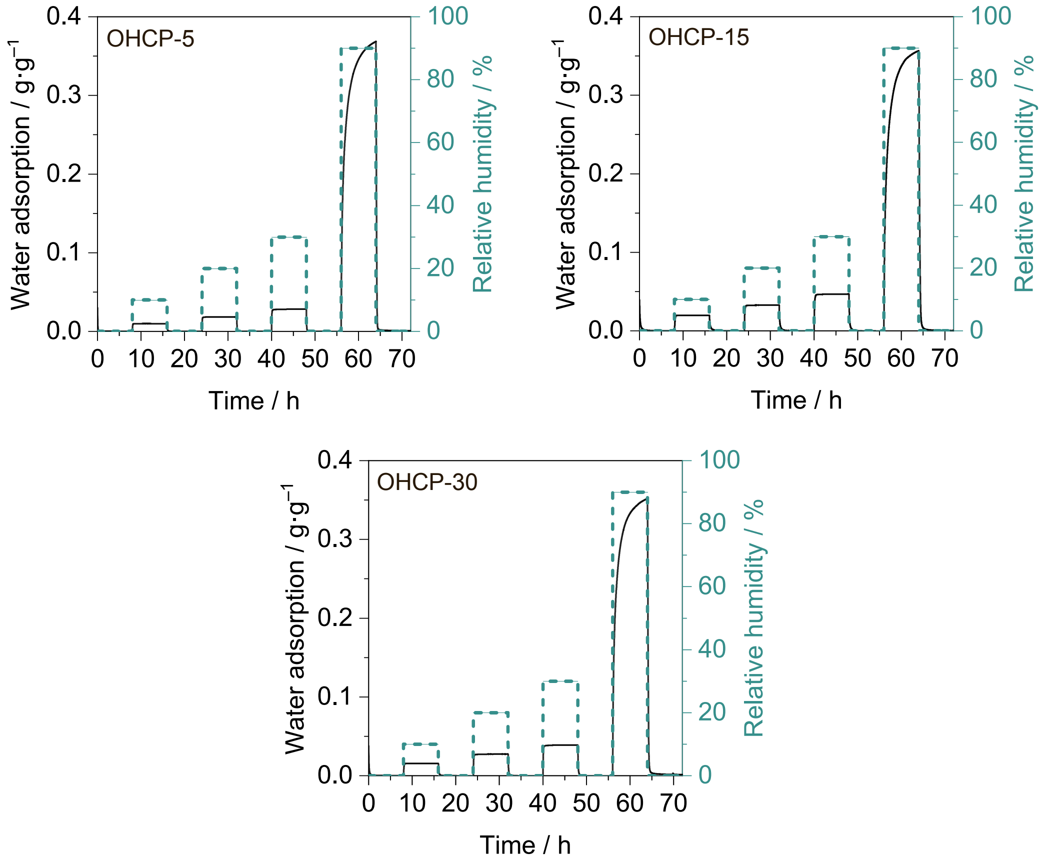

Figure S7. Dynamic vapor sorption–desorption at 10%, 20%, 30%, and 90% RH for OHCP-5, OHCP-15, and OHCP-30.

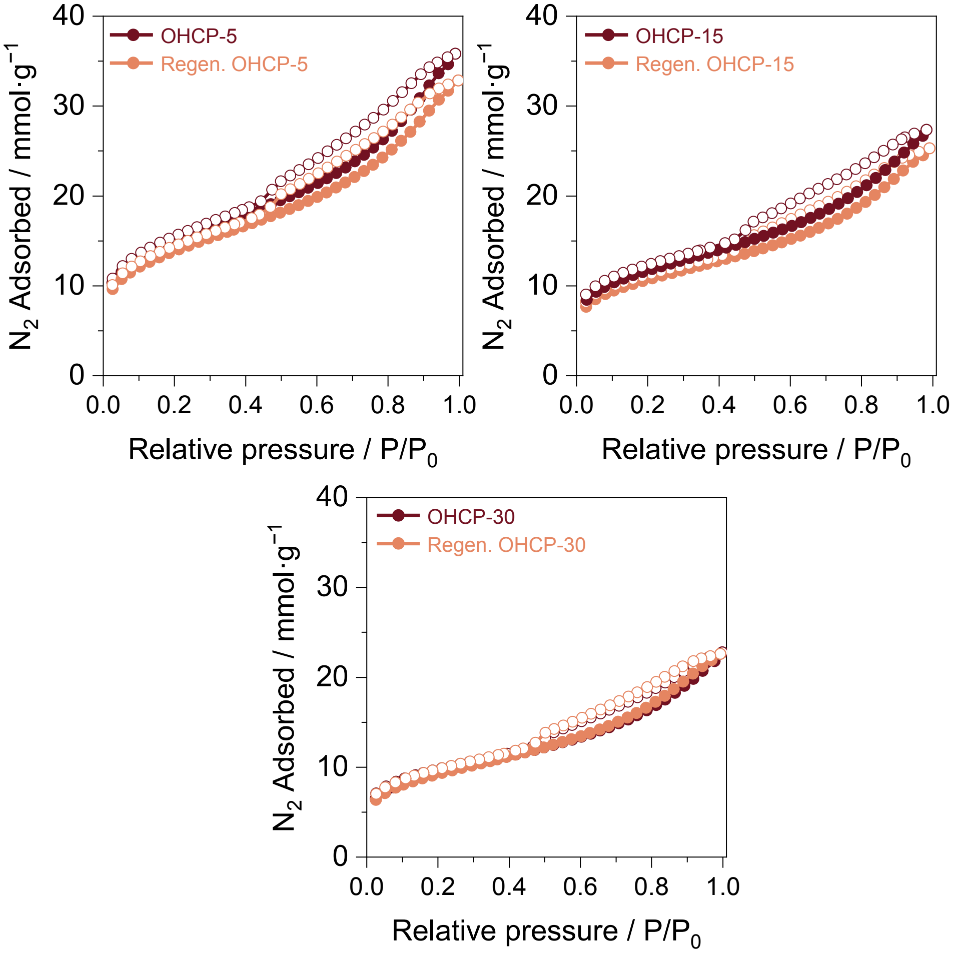

Figure S8. N_2 sorption isotherms of all OHCPs before and after (regenerated OHCP) heat cycling. 10 cycles were performed using a 90 °C desorption step for 1 h in each cycle.

Table S5. Summary of textural properties of all OHCPs before and after (regenerated OHCP) heat cycling. 10 cycles were performed using a 90 °C desorption step for 1 h in each cycle. Data includes BET specific surface area, SSA_{BET}, volume of micropores, V_{MICRO}, and total pore volume, V_{TOT} (n = 3).

Sample	SSA_{BET} ($m^2·g^{-1}$)	V_{MICRO} ($cm^3·g^{-1}$)	V_{TOT} ($cm^3·g^{-1}$)
OHCP-5	1029 ± 127	0.15 ± 0.01	1.05 ± 0.15
Regen. OHCP-5	1058 ± 29	0.14 ± 0.01	1.08 ± 0.02
OHCP-15	860 ± 152	0.14 ± 0.03	0.89 ± 0.14
Regen. OHCP-15	827 ± 72	0.11 ± 0.01	0.83 ± 0.03
OHCP-30	681 ± 109	0.10 ± 0.01	0.73 ± 0.12
Regen. OHCP-30	711 ± 87	0.09 ± 0.01	0.77 ± 0.10
OHCP-60	462 ± 68	0.06 ± 0.02	0.52 ± 0.07
Regen. OHCP-60	476 ± 16	0.06 ± 0.01	0.54 ± 0.02

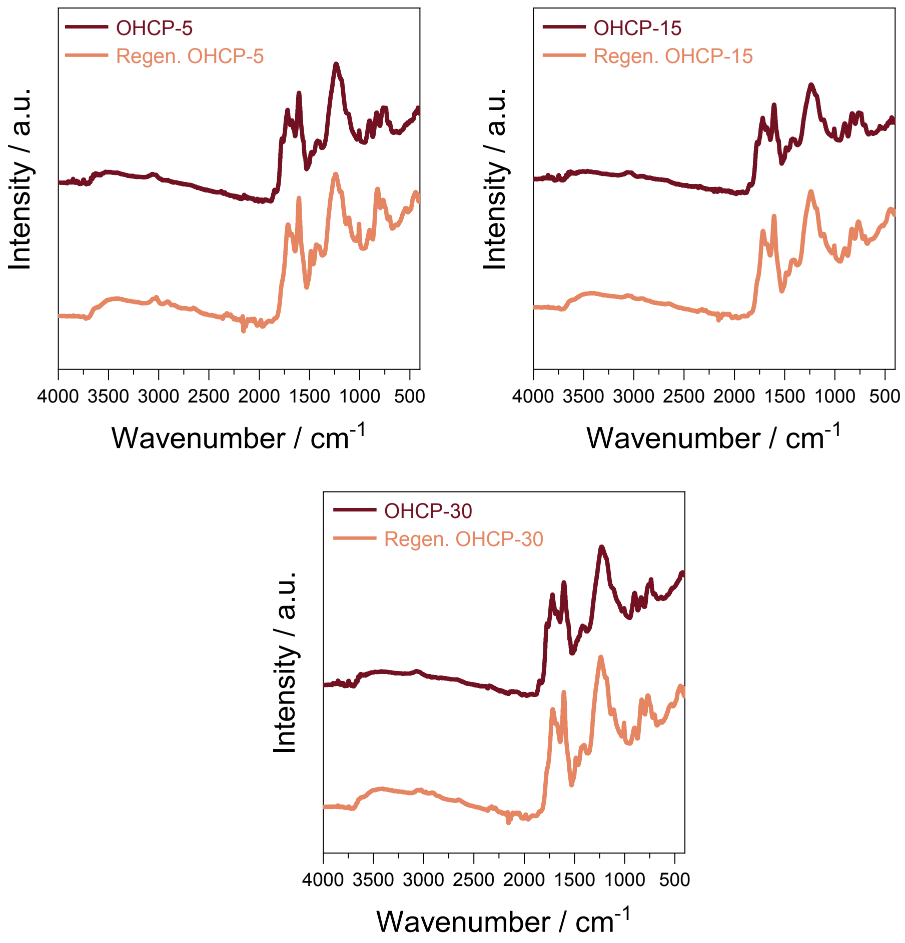

Figure S9. FTIR spectra of all OHCPs before and after (regenerated OHCP) heat cycling. 10 cycles were performed using a 90 °C desorption step for 1 h in each cycle.

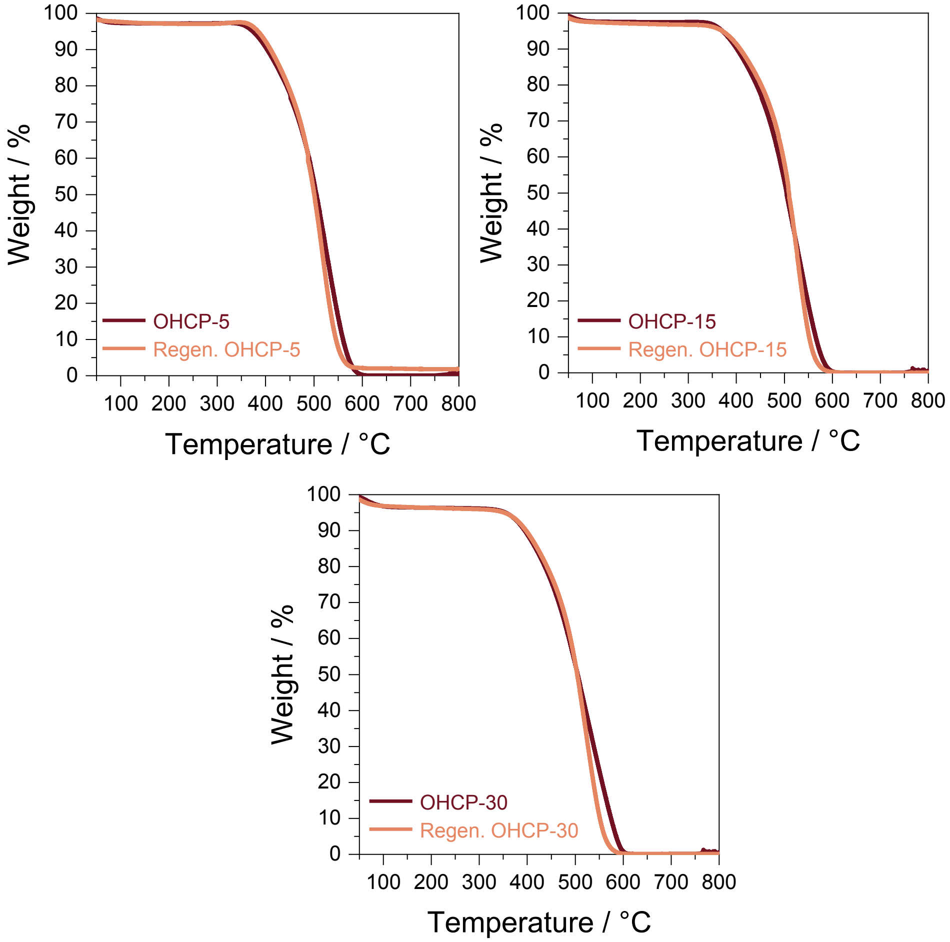

Figure S10. Thermogravimetric curves for all OHCPs before and after (regenerated OHCP) heat cycling. 10 cycles were performed using a 90 °C desorption step for 1 h in each cycle. OHCPs were heated under airflow (100 mL·min^{-1}) to 800 °C at a rate of 10 °C/min.

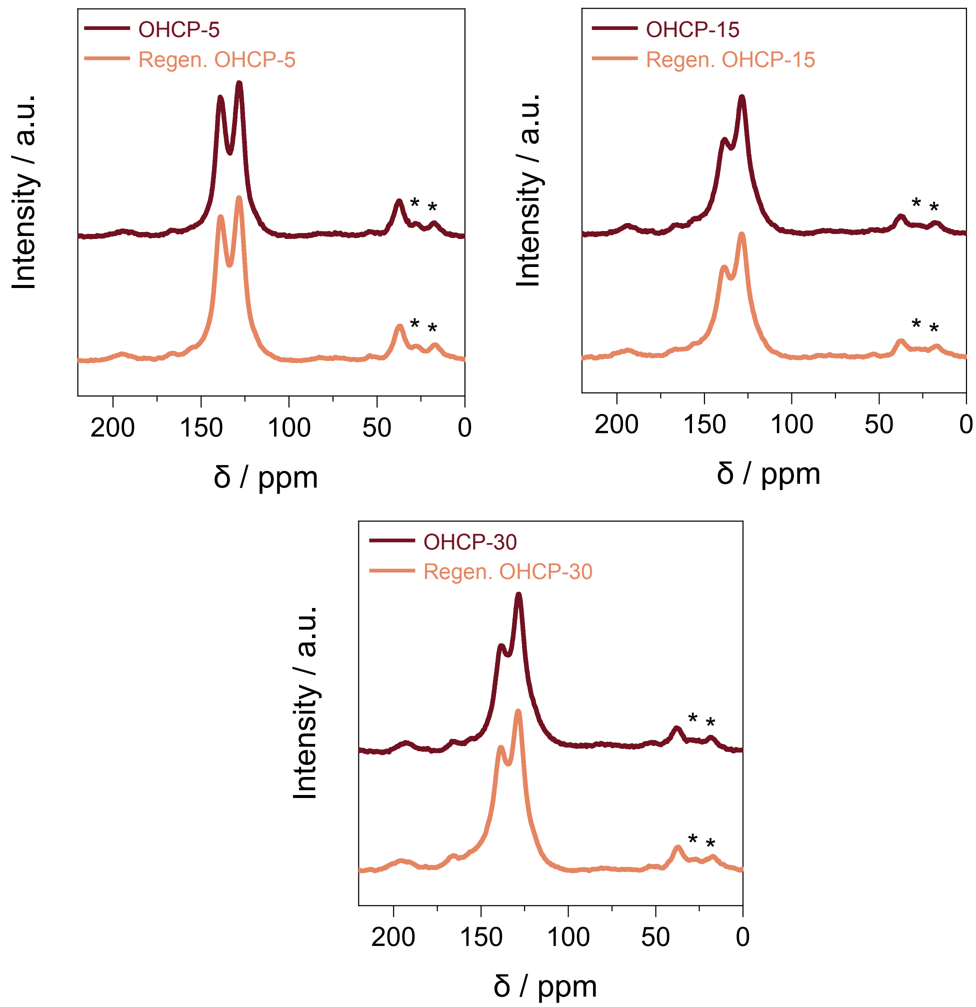

Figure S11. ^{13}C CP/MAS solid-state NMR spectra for all OHCPs before and after (regenerated OHCP) heat cycling. 10 cycles were performed using a 90 °C desorption step for 1 h in each cycle. * Represents spinning side bands.

References

[1] Biswal, B. P.; Kandambeth, S.; Chandra, S.; Shinde, D. B.; Bera, S.; Karak, S.; Garai, B.; Kharul, U. K.; Banerjee, R. Pore Surface Engineering in Porous, Chemically Stable Covalent Organic Frameworks for Water Adsorption. *J. Mater. Chem. A* **2015**, *3* (47), 23664–23669. https://doi.org/10.1039/C5TA07998E.

[2] Schweng, P.; Li, C.; Guggenberger, P.; Kleitz, F.; Woodward, R. T. A Sulfonated Covalent Organic Framework for Atmospheric Water Harvesting. *ChemSusChem*, **2024** accepted article, e202301906. https://doi.org/10.1002/cssc.202301906.

[3] Sun, C.; Zhu, Y.; Shao, P.; Chen, L.; Huang, X.; Zhao, S.; Ma, D.; Jing, X.; Wang, B.; Feng, X. 2D Covalent Organic Framework for Water Harvesting with Fast Kinetics and Low Regeneration Temperature. *Angew. Chem. Int. Ed.* **2023**, *62* (11), e202217103. https://doi.org/10.1002/anie.202217103.

[4] Chen, L.-H.; Han, W.-K.; Yan, X.; Zhang, J.; Jiang, Y.; Gu, Z.-G. A Highly Stable Ortho-Ketoenamine Covalent Organic Framework with Balanced Hydrophilic and Hydrophobic Sites for Atmospheric Water Harvesting. *ChemSusChem* **2022**, *15* (24), e202201824. https://doi.org/10.1002/cssc.202201824.

[5] Liu, Y.; Han, W.-K.; Chi, W.; Fu, J.-X.; Mao, Y.; Yan, X.; Shao, J.-X.; Jiang, Y.; Gu, Z.-G. One-Dimensional Covalent Organic Frameworks with Atmospheric Water Harvesting for Photocatalytic Hydrogen Evolution from Water Vapor. *Appl. Catal. B.* **2023**, *338*, 123074. https://doi.org/10.1016/j.apcatb.2023.123074.

[6] Nguyen, H. L.; Hanikel, N.; Lyle, S. J.; Zhu, C.; Proserpio, D. M.; Yaghi, O. M. A Porous Covalent Organic Framework with Voided Square Grid Topology for Atmospheric Water Harvesting. *J. Am. Chem. Soc.* **2020**, *142* (5), 2218–2221. https://doi.org/10.1021/jacs.9b13094.

[7] Zhu, Q.; Wang, X.; Clowes, R.; Cui, P.; Chen, L.; Little, M. A.; Cooper, A. I. 3D Cage COFs: A Dynamic Three-Dimensional Covalent Organic Framework with High-Connectivity Organic Cage Nodes. *J. Am. Chem. Soc.* **2020**, *142* (39), 16842–16848. https://doi.org/10.1021/jacs.0c07732.

[8] Grunenberg, L.; Savasci, G.; Emmerling, S. T.; Heck, F.; Bette, S.; Cima Bergesch, A.; Ochsenfeld, C.; Lotsch, B. V. Postsynthetic Transformation of Imine- into Nitrone-Linked Covalent Organic Frameworks for Atmospheric Water Harvesting at Decreased Humidity. *J. Am. Chem. Soc.* **2023**, *145* (24), 13241–13248. https://doi.org/10.1021/jacs.3c02572.

[9] Nguyen, H. L.; Gropp, C.; Hanikel, N.; Möckel, A.; Lund, A.; Yaghi, O. M. Hydrazine-Hydrazide-Linked Covalent Organic Frameworks for Water Harvesting. *ACS Cent. Sci.* **2022**, *8* (7), 926–932. https://doi.org/10.1021/acscentsci.2c00398.

[10] Stegbauer, L.; Hahn, M. W.; Jentys, A.; Savasci, G.; Ochsenfeld, C.; Lercher, J. A.; Lotsch, B. V. Tunable Water and CO_2 Sorption Properties in Isostructural Azine-Based Covalent Organic Frameworks through Polarity Engineering. *Chem. Mater.* **2015**, *27* (23), 7874–7881. https://doi.org/10.1021/acs.chemmater.5b02151.

[11] Mou, K.; Meng, F.; Zhang, Z.; Li, X.; Li, M.; Jiao, Y.; Wang, Z.; Bai, X.; Zhang, F. Pyridazine-Promoted Construction of Vinylene-Linked Covalent Organic Frameworks with Exceptional Capability of Stepwise Water Harvesting. *Angew. Chem. Int. Ed.* **2024**, *63* (34), e202402446. https://doi.org/10.1002/anie.202402446.

[12] Park, K.; Lee, K.; Kim, H.; Ganesan, V.; Cho, K.; Jeong, S. K.; Yoon, S. Preparation of Covalent Triazine Frameworks with Imidazolium Cations Embedded in Basic Sites and Their Application for CO_2 Capture. *J. Mater. Chem. A* **2017**, *5* (18), 8576–8582. https://doi.org/10.1039/C6TA11226A.

[13] Huang, J.; Yang, Y.; Chen, L.; Zhang, Z.; Xiang, S. Atmospheric Water Harvesting in Microporous Organic Polymers Constructed from Trazine and Benzimidazole Units. *ZAAC* **2023**, *649* (19), e202300167. https://doi.org/10.1002/zaac.202300167.

[14] Schweng, P.; Mayer, F.; Galehdari, D.; Weiland, K.; Woodward, R. T. A Robust and Low-Cost Sulfonated Hypercrosslinked Polymer for Atmospheric Water Harvesting. *Small* **2023**, *19* (50), 2304562. https://doi.org/10.1002/smll.202304562.

[15] Byun, Y.; Coskun, A. Epoxy-Functionalized Porous Organic Polymers via the Diels–Alder Cycloaddition Reaction for Atmospheric Water Capture. *Angew. Chem. Int. Ed.* **2018**, *57* (12), 3173–3177. https://doi.org/10.1002/anie.201800380.
